# Monoclonal antibody MA454 reveals a heterogeneous expression pattern of MAGE-1 antigen in formalin-fixed paraffin embedded lung tumours

**DOI:** 10.1054/bjoc.2000.1291

**Published:** 2000-07-24

**Authors:** A A Jungbluth, E Stockert, Y-T Chen, D Kolb, K Iversen, K Coplan, B Williamson, N Altorki, K J Busam, L J Old

**Affiliations:** Ludwig Institute for Cancer Research at Memorial Sloan-Kettering Cancer Center, 1275 York Avenue, New York, NY 10021, USA; Department of Pathology, Department of Thoracic Surgery, New York Presbyterian Hospital-Weill Cornell Medical Center, 1300 York Avenue, New York, NY 10021, USA; Department of Pathology, Memorial Sloan-Kettering Cancer Center, 1275 York Avenue, New York, NY 10021, USA

**Keywords:** MAGE-1 antigen, monoclonal antibody MA454

## Abstract

Cancer/testis (CT) antigens such as those encoded by the MAGE-gene family are expressed in a wide variety of malignant neoplasms. In normal tissues, expression is generally restricted to testis. Current knowledge of the expression pattern of CT antigens is mainly based on mRNA analysis. Little is known about actual protein expression. We previously developed MA454, a monoclonal antibody (mAb) to MAGE-1 recombinant protein. By employing antigen retrieval techniques, we show that MA454 is reactive on formalin-fixed paraffin-embedded tissues. Immunohistochemical (IHC) analysis of a normal tissue panel revealed staining solely in germ cells of testes. A series of 59 lung tumours was co-typed for MAGE-1 expression by RT–PCR and by immunohistochemistry with MA454. MA454 was positive in 19/59 cases (32%). MAGE-1 mRNA was found in 17 of the 54 cases (32%) available for RT–PCR. Of the 19 MA454-reactive tumours, 15 showed a highly heterogeneous pattern of expression. The other 4 MA454 positive cases revealed immunoreactivity in >25% of tumour areas. Of the 53 cases typed for both, mRNA and protein expression, 48 co-typed whereas 5 cases were discrepant, a likely consequence of heterogeneous MAGE-1 expression. The predominantly focal expression of MAGE-1 suggests that this antigen might not be sufficient as a sole target for immunotherapeutic approaches. © 2000 Cancer Research Campaign


*British Journal of Cancer* (2000) **83**(4), 493–497
© 2000 Cancer Research Campaign
doi: 10.1054/ bjoc.2000.1291, available online at http://www.idealibrary.com on IDEAL®

### Monoclonal antibody MA454 reveals a heterogeneous expression pattern of MAGE-1 antigen in formalin-fixed paraffin embedded lung tumours

**AA Jungbluth[1], E Stockert[1], Y-T Chen[2], D Kolb[1], K Iversen[1], K Coplan[1], B Williamson[1], N Altorki[3], KJ Busam[4] and LJ Old[1]**

[1]Ludwig Institute for Cancer Research at Memorial Sloan-Kettering Cancer Center, 1275 York Avenue, New York, NY 10021, USA; [2]Department of Pathology and [3]Department of Thoracic Surgery, New York Presbyterian Hospital-Weill Cornell Medical Center, 1300 York Avenue, New York, NY 10021, USA; [4]Department of Pathology, Memorial Sloan-Kettering Cancer Center, 1275 York Avenue, New York, NY 10021, USA

**Summary** Cancer/testis (CT) antigens such as those encoded by the MAGE-gene family are expressed in a wide variety of malignant neoplasms. In normal tissues, expression is generally restricted to testis. Current knowledge of the expression pattern of CT antigens is mainly based on mRNA analysis. Little is known about actual protein expression. We previously developed MA454, a monoclonal antibody (mAb) to MAGE-1 recombinant protein. By employing antigen retrieval techniques, we show that MA454 is reactive on formalin-fixed paraffin-embedded tissues. Immunohistochemical (IHC) analysis of a normal tissue panel revealed staining solely in germ cells of testes. A series of 59 lung tumours was co-typed for MAGE-1 expression by RT–PCR and by immunohistochemistry with MA454. MA454 was positive in 19/59 cases (32%). MAGE-1 mRNA was found in 17 of the 54 cases (32%) available for RT–PCR. Of the 19 MA454-reactive tumours, 15 showed a highly heterogeneous pattern of expression. The other 4 MA454 positive cases revealed immunoreactivity in >25% of tumour areas. Of the 53 cases typed for both, mRNA and protein expression, 48 co-typed whereas 5 cases were discrepant, a likely consequence of heterogeneous MAGE-1 expression. The predominantly focal expression of MAGE-1 suggests that this antigen might not be sufficient as a sole target for immunotherapeutic approaches. © 2000 Cancer Research Campaign

**Keywords:** MAGE-1 antigen; monoclonal antibody MA454

Cancer/testis (CT) antigens are a recently recognized category of tumour antigens that are expressed in a variety of malignant neoplasms, but silent in normal tissues except testis. For this reason, CT antigens appear to be ideal targets for immunotherapy of human cancer (Boon and Old, 1997; Van den Eynde and Boon, 1997). There are now ten genes or gene families coding for antigens with these characteristics (De Plaen et al, 1994; Lurquin et al, 1997; Sahin et al, 1997; Chen and Old, 1998; Lucas et al, 1998). MAGE genes, the first family of genes coding for CT antigens to be recognized, code for tumour products with a characteristic pattern of CT expression, and MAGE-1 was the first MAGE gene identified (Van der Bruggen et al, 1991; Traversari et al, 1992). Current knowledge of CT antigen expression is mainly based on the analysis of mRNA, and little is known about actual protein expression of these antigens. We previously generated a monoclonal antibody, designated MA454, to MAGE-1 recombinant protein (Chen et al, 1994). Although MA454 identified the MAGE gene product in western blots and ELISA, it could not detect the antigen in tissue specimens using techniques available. Recent advances in antigen retrieval techniques have prompted us to reanalyse the immunohistochemical reactivity of MA454.

In previous studies, MAGE-1 mRNA was found to be expressed in a high percentage of pulmonary neoplasms (Van den Eynde and Van der Bruggen, 1997). The present study assesses the reactivity of mAb MA454 with normal tissues and with a series of lung neoplasms. Furthermore, we compared MA454 reactivity with expression of MAGE-1 mRNA by RT–PCR.

*Received 30 November 1999*
*Revised 7 March 2000*
*Accepted 7 March 2000*

*Correspondence to:* AA Jungbluth

### MATERIALS AND METHODS

### Tissues

Tissues were obtained from the Departments of Pathology of Memorial Sloan-Kettering Cancer Center and New York Hospital/Cornell University Medical School. The specimens consisted of O.C.T.-mounted (Tissue Tek, Torrance, CA), snap-frozen tissue samples and formalin-fixed, paraffin-embedded tissue blocks. Five μm sections were cut from frozen and paraffin blocks and were applied to histology slides for immunohistochemistry (Superfrost Plus, Fisher Scientific, Pittsburgh, PA). A panel of normal tissues and a series of lung neoplasms were tested as indicated in Table 1 and Table 2. The lung tumours were also analyzed by RT–PCR for the presence of MAGE-1 mRNA.

### Immunohistochemistry

The generation of MA454, a murine IgG1 mAb to the MAGE-1 recombinant protein, was previously described (Chen et al, 1994). Initial titration and reactivity assessments were done on frozen and paraffin testicular specimens. Testis was also used as a positive control tissue in subsequent assays. For frozen tissues, different fixation protocols such as acetone and formaldehyde solutions

**Table 1** Immunohistochemistry of MA454 in formalin-fixed paraffin-embedded normal tissues

| Tissue | MA454 |
|---|---|
| Oesophagus | – |
| Stomach | – |
| Duodenum | – |
| Small intestine | – |
| Colon | – |
| Appendix | – |
| Liver | – |
| Pancreas | – |
| Parotid gland | – |
| Kidney | – |
| Ureter (renal pelvis) | – |
| Urinary bladder | – |
| Prostate | – |
| Testis | positive |
| Uterus (cervix/endometrium) | – |
| Fallopian tube | – |
| Ovary | – |
| Breast | – |
| Placenta | – |
| Skeletal muscle | – |
| Thyroid gland | – |
| Adrenal gland | – |
| Lymph node | – |
| Thymus | – |
| Spleen | – |
| Tonsil | – |
| Heart | – |
| Lung | – |
| Skin | – |
| Peripheral nerve | – |

were tested. Staining of paraffin sections was tested without any pretreatment, as well as with heat-based antigen retrieval methods using citrate buffer (10 mM, pH 6.0), EDTA buffer (1 mM, pH 8.0) and commercial retrieval solutions like DAKO-TRS (DAKO, Carpintera, CA), and DAKO hipH. The primary antibody was detected with a biotinylated horse anti-mouse-secondary reagent (1:200; Vector Laboratories, Burlingham, CA) followed by an avidin-biotin-complex system (Vector) using diaminobenzidine tetrahydrochloride (DAB, Biogenex, San Ramon, CA) as a chromogen. The extent of immunohistochemical reactivity in tumour tissues was estimated by light microscopy and graded according to the number of immunoreactive cells in 25% increments: 'focal' indicating staining of single cells or small clusters of cells (not more than 5% cells stained); + = <25%, ++ = 25–50%, +++ = 50–75%, and ++++ = >75% of cells stained. A weak staining intensity was indicated by 'w'. Control slides consisted of testis tissue as a positive control; negative control slides were incubated with buffer instead of MA454.

### RT–PCR

In order to determine the specificity of MA454, lung tumours were typed for MAGE-1 by RT–PCR and the results were compared with MA454 immunohistochemical staining. RT–PCR was done as previously described (Chen et al, 1994). Briefly, total RNA was extracted from 20 μm sections of corresponding frozen tissue blocks. Testicular tissue was used as a positive control tissue. RNA was reverse transcribed into cDNA and PCR-amplified with AmpliTaqGold (Perkin Elmer, Norwalk, CT) for 30 cycles in a

**Table 2** Immunoreactivity of MA454 with formalin-fixed paraffin-embedded lung tumours

| Case | Diagnosis | MAGE-1 mRNA | MA45 4-immunoreactivity[a] |
|---|---|---|---|
| 1 | LCC | – | – |
| 2 | ADC | +[b] | + |
| 3 | LCC | +[b] | foc. |
| 4 | SQCC | – | foc. w |
| 5 | ADC | +[b] | foc. |
| 6 | ADC | +[b] | + |
| 7 | SQCC | – | – |
| 8 | ADC | – | – |
| 9 | ADC | – | – |
| 10 | ADC | – | foc. |
| 11 | ADC | – | – |
| 12 | LCC | + | foc. |
| 13 | ADC | – | – |
| 14 | ADC | + | – |
| 15 | ADC | + | foc. |
| 16 | SQCC | + | – |
| 17 | ADC | + | + w |
| 18 | LCC | – | – |
| 19 | ADC | – | – |
| 20 | ADC, BA | – | – |
| 21 | carcinosarcoma | +[b] | + |
| 22 | ADC | – | – |
| 23 | ADC, papillary | – | – |
| 24 | SQCC | +[b] | + w |
| 25 | ADC with focal SQCC differentiation | – | – |
| 26 | LCC | – | foc. |
| 27 | SQCC | – | – |
| 28 | ADC, BA | – | – |
| 29 | ADC | + | ++++ |
| 30 | ADC | – | – |
| 31 | ADC | – | – |
| 32 | SQCC | – | – |
| 33 | SQCC | n.a. | ++ |
| 34 | ADC, BA | n.a. | – |
| 35 | ADC | – | – |
| 36 | ADC | – | – |
| 37 | SQCC | + | ++ |
| 38 | SQCC | n.a. | – |
| 39 | ADC | – | – |
| 40 | SQCC | + | foc. |
| 41 | ADC | + | + |
| 42 | ADC, BA | – | – |
| 43 | ADC | + | ++ |
| 44 | ADC | – | – |
| 45 | LCC | – | – |
| 46 | LCC | – | – |
| 47 | ADC | n.a. | – |
| 48 | ADC | – | – |
| 49 | ADC | – | – |
| 50 | SQCC | – | – |
| 51 | ADC | – | – |
| 52 | LCC | + | + |
| 53 | SQCC | – | – |
| 54 | ADC | – | – |
| 55 | LCC | – | – |
| 56 | ADC | – | – |
| 57 | ADC, clear cell | – | – |
| 58 | ADC | n.a. | – |
| 59 | ADC, papillary | – | – |

[a]grading of immunohistochemical tumour staining as follows: foc.: immunoreactivity approximately <5%, +: 5–25%, ++: 25–50%, +++:5 0–75%, ++++: >75%, 'w' weak immunostaining, [b]sequence analysis of RT-PCR product, n.a.: not available, LCC: large cell undifferentiated carcinoma, SQCC: squamous cell carcinoma, ADC: adenocarcinoma, ADC, BA: adenocarcinoma, bronchioalveolar type.

*© 2000 Cancer Research Campaign*

thermal cycler (Perkin Elmer) at an annealing temperature of 60°C. Oligonucleotide primers CHO-12 and CHO-14 were used to detect MAGE-1 (Brasseur et al, 1992); both primers were synthesized commercially (GIBCO, Grand Islands, NY). RT–PCR products were visualized with ethidium bromide. In order to confirm the specificity of the RT–PCR products, a sequence analysis was performed commercially (Bioresource Center, Cornell University, Ithaca, NY) for 6 representative tissues (cases #2, #3, #5, #6, #21, #24).

### RESULTS

MA454 showed poor reactivity in frozen tissue sections. Reproducible, strong immunoreactivity was observed in formalin-fixed, paraffin-embedded standard archival tissue when using an antigen retrieval technique. Hence, further assays were done on formalin-fixed paraffin-embedded tissues. The best staining was achieved after heating sections at 90°C in EDTA for 30 minutes. A biotin/avidin blocking kit (Vector, Elite) was used to suppress the background staining due to endogenous biotin activity.

Table 1 summarizes the immunohistochemical staining properties of MA454 in normal tissues. No staining could be observed in any normal tissue except testis. The testicular immunoreactivity was confined to components within the seminiferous tubules and staining was restricted to germ cells. Spermatogonia showed strong immunoreactivity, with a lesser degree of staining in primary spermatocytes. A consistent strong cytoplasmic staining of spermatogonia was observed. Germ cells at later maturation stages, e.g. spermatocytes, showed a more variable labelling of the cytoplasm. This staining varied from none to mostly faint and occasionally moderate in spermatocytes, depending on the concentration of MA454 and the particular specimen used. As with spermatogonia, staining of spermatocytes was cytoplasmic with no significant nuclear reactivity. Spermatids, Sertoli cells, and intertubular tissue components such as Leydig cells remained immunonegative (Figure 1A, B).

Table 2 summarizes the results of immunohistochemical staining and RT–PCR analysis of the lung tumours. Fifty-nine cases were available for immunohistochemical evaluation. All except one case of carcinosarcoma were non-small cell lung carcinomas. From 54 cases, fresh tissue for RT–PCR analysis was also available. Immunohistochemistry revealed immunopositivity with MA454 in 19/59 cases (32%). However, in the vast majority of tumours, MA454 revealed a predominantly heterogeneous reactivity pattern (Fig. 1C–F), with ‘focal’ immunoreactivity (8 cases) or immunoreactivity in <25% of the tumour (7 cases). These tissues showed MA454 positive single tumour cells or small tumour nests (Fig. 1C, F). Only in 4 tissues was staining seen in wider areas (>25%) of the tumour. One of these 4 cases (Fig. 1D) revealed homogenous immunoreactivity in all neoplastic areas (‘++++’). The cellular staining pattern was cytoplasmic and no nuclear staining was observed (Fig. 1E). Among 37 adenocarcinoma cases in our series, 9 were immunoreactive. Of the 12 squamous cell carcinoma and 9 large cell undifferentiated carcinoma cases, 5 and 4 cases were MA454 positive, respectively. The carcinosarcoma was also MA454 positive. The 4 cases with more widespread MA454-reactivity were two squamous cell carcinomas and two adenocarcinomas. In case 43, two blocks were available for analysis: while one block showed staining in more than 25% of the tumour, the other block revealed a focal staining. RT–PCR showed the presence of MAGE-1 mRNA in 17/54 cases (32%). The sequencing analysis of the RT–PCR products confirmed the presence of MAGE-1 mRNA. The results of RT–PCR typing matched IHC staining in 49 of the 54 cases (91%). Of the 5 cases that did not co-type with MA454 expression, 3 were MAGE-1 mRNA negative and IHC positive, whereas two cases were mRNA positive and MA454 negative. All MA454 positive and MAGE-1 RT–PCR negative cases showed focal immunoreactivity.

### DISCUSSION

A number of serological reagents for the evaluation of MAGE protein expression has been generated (Chen et al, 1994; Schultz-Thater et al, 1994; Kocher et al, 1995; Takahashi et al, 1995; Carrel et al, 1996; Jurk et al, 1998). One of the most intensely studied MAGE reagents is 57B, a mAb generated against MAGE-3 recombinant protein (Fischer et al, 1997; Hofbauer et al, 1997; Cheville and Roche, 1999). Although initially thought to have specificity for MAGE-3, subsequent analysis with COS cells transfected with individual MAGE genes, has shown that 57B detects MAGE-1, -2, -3, -4, -6, and -12 (M Godelaine, personal communication). With regard to MAGE-1, several monoclonal antibodies have developed. Mab 6C1 reacts with MAGE-10 as well as MAGE-1 (Carrel et al, 1996; Rimoldi et al, 1999), and the fine specificity of 77B has not been reported (Gudat et al, 1996; Zuber et al, 1997). MA454, the anti-MAGE-1 mAb used in this analysis, originally did not show cross-reactivity with other MAGE proteins (Chen et al, 1994). Carrel et al (1996) describing the generation of their anti-MAGE mAb 6C1 included MA454 and did not find any indication of MA454 cross-reacting with other proteins but MAGE-1. Recently, an immunohistochemical analysis of a panel of MAGE-transfected cells, also confirmed that MA454 was specific for MAGE-1 and did not react with MAGE-2, -3, -4, -6, -8, and -9 to -12 (M. Godelaine, manuscript in preparation).

In the initial analysis of MA454, no immunoreactivity could be shown using frozen tumour specimens (Chen et al, 1994). However, when antigen retrieval techniques are used, MA454 shows strong reactivity on formalin-fixed paraffin-embedded specimens. Antigen retrieval has only recently become an important tool for immunohistochemistry and is now employed as a standard procedure in pathology (Shi et al, 1996). At this point, we can only speculate about reasons for recovering MAGE-1 reactivity after antigen retrieval. Possibly the structure of the denatured MAGE-1 recombinant protein used for immunization resembles the antigen present in formalin-fixed, paraffin-embedded tissues after antigen retrieval more closely than its acetone-fixed counterpart. Another possibility is that antigen retrieval exposes epitopes not accessible in acetone-fixed specimens.

In the present study, the immunoreactivity of MA454 with normal tissues closely correlated with the known MAGE-1 mRNA expression pattern (De Plaen et al, 1994) i.e. only testis was immunoreactive. With regard to spermatogenic cells, MA454 staining contrasts with the pattern seen with mAb 57B, anti-MAGE-4 mAb R5 and a MAGE-1 polyclonal reagent (Takahashi et al, 1995; Itoh et al, 1996; Jungbluth et al, 2000). The latter three mAbs give a strong nuclear staining, while MA454 is confined to the cytoplasm. The staining of later stage germ cells, i.e. spermatocytes, varied, ranging from mostly faint and moderate to no staining, depending on MA454 concentrations and individual

© 2000 Cancer Research Campaign

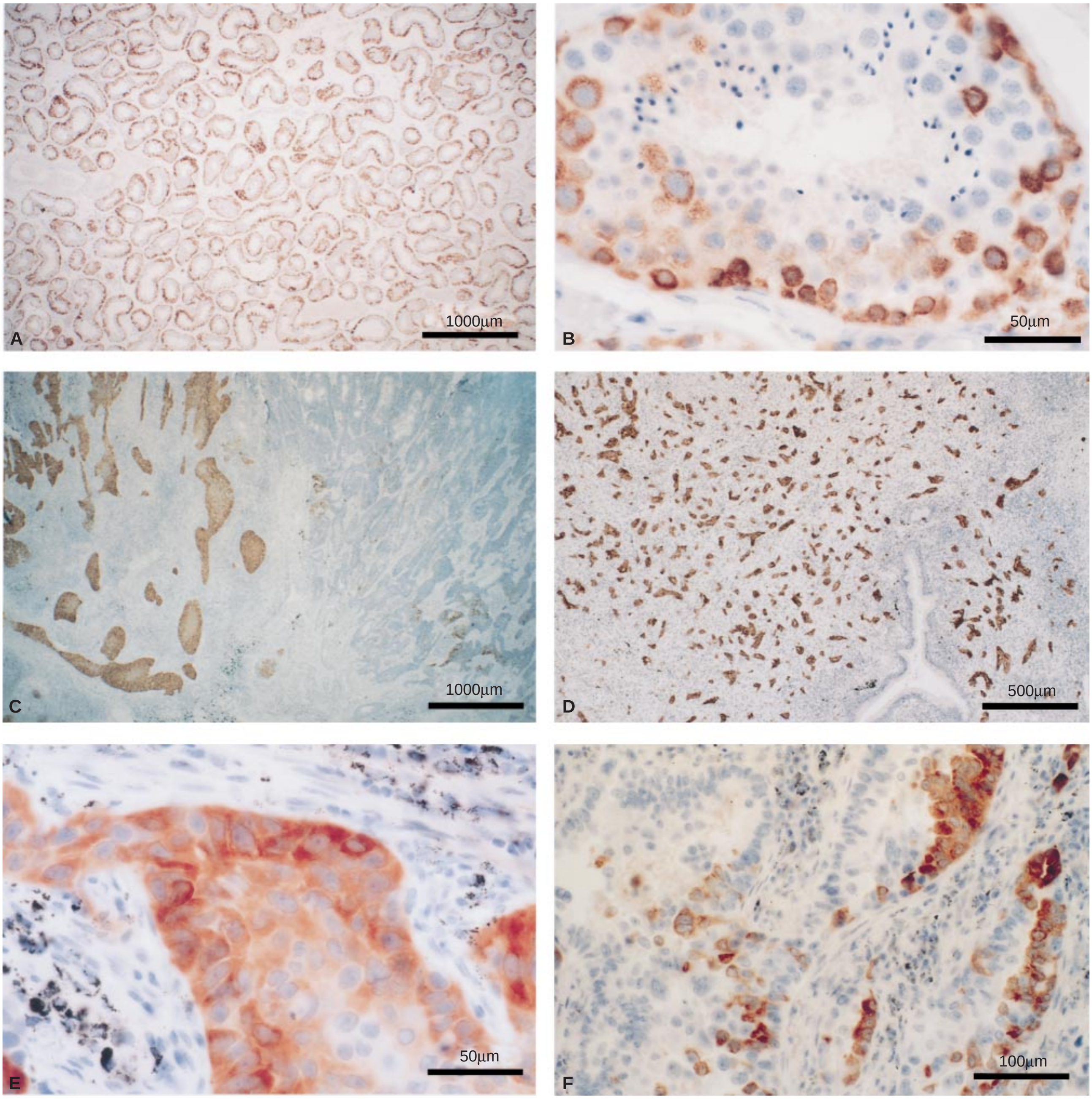

**Figure 1** Immunohistochemical staining with MA454, normal testis (**A**, **B**) and lung carcinomas (**C–F**). Avidin-biotin technique, chromogen DAB, haematoxylin counterstain. (**A**) Normal testis. Staining of germ cells within seminiferous tubules. Negative interstitial tissue. (**B**) Seminiferous tubule. Intensely stained spermatogonia and variable staining of spermatocytes. Spermatids and Sertoli cells not stained. (**C**) Squamous cell carcinoma (case 33). An area of MA454 reactivity next to an immunonegative tumour area. (**D**) Adenocarcinoma (case 29). Homogeneous immunoreactivity in all tumour areas. (**E**) Squamous cell carcinoma (case 37). Intense cytoplasmic staining, negative nuclei. (**F**) Adenocarcinoma (case 41). Heterogeneous staining pattern, intensely immunopositive cells next to negative cells specimens. Thus, MAGE-1 protein is present in early stages of germ cell maturation, with the highest level of expression in spermatogonia and decreasing expression levels inversely paralleling germ cell maturation. As little is known about the biological function of the MAGE-proteins, an interpretation of these different staining patterns is difficult. The expression pattern of different MAGE proteins suggests that each gene plays a different role in different stages of germ cell maturation.

In this series of lung neoplasms, frozen and paraffin tissues were available for most cases and side by side immunohistochemistry and RT–PCR typing could be performed. Previous mRNA analyses for MAGE-1 expression of lung tumours have varied widely: 11% (Sakata, 1996), 20% (Ferlazzo et al, 1996), 35% (Weynants et al, 1994), 49% (Van Den Eynde and Van der Bruggen, 1997) or more than 60% (Fischer et al, 1997). In our series, 32% of the tumours expressed MAGE-1 by RT–PCR, and immunohistochemical staining with MA454 revealed 32% positive tumours. Overall, protein expression correlated well with the mRNA analysis, most of the RT–PCR-positive tissues showing some degree of MA454 immunoreactivity. Similar to

 © 2000 Cancer Research Campaign spermatogenic cells, staining was restricted to cytoplasm of the tumour cells and no significant nuclear immunoreactivity was observed. Cytoplasmic localization of the MAGE-1 antigen was previously shown by cell fractionation studies of cultured tumour cells using mAb MA454 (Amar-Costesec et al, 1994) and by immunofluorescence in cultured cells with mAb 77B (Schultz-Thater et al, 1994; Gudat et al, 1996).

In our immunohistochemical analysis, 4 cases showed expression in >25% of the tumour; only 1 of these gave homogeneous staining. However, most tumours showed a restricted 'focal' staining or immunoreactivity in less than 25% of the tumour. This extreme heterogeneous immunoreactivity likely explains the discrepancy between mRNA- and MA454 expression in 5 cases as due to error variations. This is supported by the fact that all discrepant MA454-positive/RT–PCR negative cases revealed only focal immunoreactivity. Due to the predominance of immunonegative areas, samples could easily contain solely non-reactive cells. Also, degradation of mRNA in tissue specimens cannot be excluded. The heterogeneity with MA454 is more pronounced than we saw in our previous study with mAb 57B (Jungbluth et al, 2000). This is probably due to the fact that 57B is essentially a polyvalent MAGE reagent. Tumour 43, a case in which 2 paraffin blocks were available for assessment, illustrates the heterogeneous expression of MAGE-1 even in a single patient. While one block showed focal reactivity, the other block revealed immunostaining in almost 50% of the tumour. A similar heterogeneity was observed in a limited number of melanoma specimens with mAb 77B (Zuber et al, 1997). Genomic demethylation has been associated with the activation of the MAGE-1 gene (De Smet et al, 1996), but how this relates to expression by some tumours and not by others is unexplained. As homogeneously expressed antigens are preferred targets for immunotherapy, MAGE-1 might not be a sufficient sole target for immunotherapeutic approaches in lung neoplasms. Though mRNA expression analyses might suggest a high percentage of positive tumours for any particular antigen, our investigation demonstrates that an immunohistochemical examination renders a different picture for protein expression and is essential for the evaluation of targets for immunotherapy.

### REFERENCES

Amar-Costesec A, Godelaine D, Stockert E, Van der Bruggen P, Beaufay H and Chen YT (1994) The tumor protein MAGE-1 is located in the cytosol of human melanoma cells. *Biochem Biophys Res Commun* **204**: 710–715

Boon T and Old LJ (1997) Cancer tumor antigens *Curr Opin Immunol* **9**: 681–683

Brasseur F, Marchand M, Vanwijck R, Herin M, Lethe B, Chomez P and Boon T (1992) Human gene MAGE-1, which codes for a tumor-rejection antigen, is expressed by some breast tumors. *Int J Cancer* **52**: 839–841

Carrel S, Schreyer M, Spagnoli G, Cerottini Jc and Rimoldi D (1996) Monoclonal antibodies against recombinant-MAGE-1 protein identify a cross-reacting 72-kDa antigen which is co-expressed with MAGE-1 protein in melanoma cells. *Int J Cancer* **67**: 417–422

Chen YT and Old LJ (1998) New paths in human cancer serology. *J Exp Med* **20**: 1163–1167

Chen YT, Stockert E, Chen Y, Garin-Chesa P, Rettig WJ, Van der Bruggen P, Boon T and Old LJ (1994) Identification of the MAGE-1 gene product by monoclonal and polyclonal antibodies. *Proc Natl Acad Sci USA* **91**: 1004–1008

Cheville JC and Roche PC (1999) MAGE-1 and MAGE-3 tumor rejection antigens in human germ cell tumors. *Mod Pathol* **12**: 974–978

De Plaen E, Arden K, Traversari C, Gaforio JJ, Szikora JP, de Smet C, Brasseur F, Van der Bruggen P, Lethé B, Lurquin B, Brasseur R, Chomez P, de Backer O, Cavanee W and Boon T (1994) Structure, chromosomal localization, and expression of 12 genes of the MAGE family. *Immunogenetics* **40**: 360–367

De Smet C, de Backer O, Faraoni I, Lurquin C, Brasseur F and Boon T (1996) The activation of human gene MAGE-1 in tumor cells is correlated with genome-wide demethylation. *Proc Natl Acad Sci* **93**: 7149–7153

Ferlazzo G, Meta M, Mesiti M, Cangemi G, Iemmo R, Quartarone G, Fecarotta E, Semino C, Pietra G and Melioli G (1996) Detection of MAGE-1, -2, and -3 messenger RNA in tissue samples derived from lung and mammary tumors. *Ann N Y Acad Sci* **784**: 448–452

Fischer C, Gudat F, Stulz P, Noppen C, Schaefer C, Zajac P, Trutmann M, Kocher T, Zuber M, Harder F, Heberer M and Spagnoli GC (1997) High expression of MAGE-3 protein in squamous-cell lung carcinoma [letter]. *Int J Cancer* **71**: 1119–1121

Gudat F, Zuber M, Durmuller U, Kocher T, Schaefer C, Noppen C and Spagnoli G (1996) The tumour-associated antigen MAGE-1 is detectable in formal in-fixed paraffin sections of malignant melanoma. *Virchows Arch* **429**: 77–81

Hofbauer GF, Schaefer C, Noppen C, Boni R, Kamarashev J, Nestle FO, Spagnoli GC and Dummer R (1997) MAGE-3 immunoreactivity in formalin-fixed, paraffin-embedded primary and metastatic melanoma: frequency and distribution. *Am J Pathol* **151**: 1549–1553

Itoh K, Hayashi A, Nakao M, Hoshino T, Seki N and Shichijo S (1996) Human tumor rejection antigens MAGE. *J Biochem* **119**: 385–390

Jungbluth AA, Busam KJ, Kolb D, Iversen K, Coplan Chen YT, Spagnoli GC and Old LJ (2000) Expression of MAGE-antigens in normal tissues and cancer. *J Exp Med* **85**: 460–465

Jurk M, Kremmer E, Schwarz U, Forster R and Winnacker E-L (1998) MAGE-11 protein is highly conserved in higher organisms and located predominantly in the nucleus. *Int J Cancer* **75**: 762–766

Kocher T, Schultz-Thater E, Gudat F, Schaefer C, Casorati G, Juretic A, Willimann T, Harder F. Heberer M and Spagnoli GC (1995) Identification and intracellular location of MAGE-3 gene product. *Cancer Res* **55**: 2236–2239

Lucas S, De Smet C, Arden KC, Viars CS, Lethé B, Lurquin C and Boon T (1998) Identification of a new MAGE gene with tumor-specific expression by representational difference analysis. *Cancer Res* **58**: 743–752

Lurquin C, de Smet C, Brasseur F, Muscatelli F, Martelange V, de Plaen E, Brasseur R, Monaco AP and Boon T (1997) Two members of the human MAGE-B gene family located in Xp21.3 are expressed in tumors of various histological origins. *Genomics* **46**: 397–408

Rimoldi D, Salvi S, Reed D, Coulie P, Jongeneel VC, De Plaen E, Brasseur F, Rodriguez AM, Boon T and Cerottini JC (1999) cDNA and protein characterization of human MAGE-10. *Int J Cancer* **82**: 901–907

Sahin U, Tureci O, Pfreundschuh M (1997) Serological identification of human tumor antigens. *Curr Opin Immunol* **9**: 709–716

Sakata M (1996) Expression of MAGE gene family in lung cancers. *Kurume Med J* **43**: 55–61

Schultz-Thater E, Juretic A, Dellabona P, Lüscher U, Siegrist W, Harder F, Heberer M, Zuber M and Spagnoli GC (1994) MAGE-1 gene product is a cytoplasmic protein. *Int J Cancer* **59**: 435–439

Shi SR, Cote RJ and Taylor CR (1996) Antigen retrieval immunohistochemistry: past, present, and future. *Appl Immunohistochem* **4**: 144–166

Takahashi K, Shichijo S, Noguchi M, Hirohata M and Itoh K (1995) Identification of MAGE-1 and MAGE-4 proteins in spermatogonia and primary spermatocytes of testis. *Cancer Res* **55**: 3478–3482

Traversari C, van der Bruggen P, Van den Eynde B, Hainaut P, Lemoine C, Ohta N, Old L and Boon T (1992) Transfection and expression of a gene coding for a human melanoma antigen recognized by autologous cytolytic T lymphocytes. *Immunogenetics* **35**: 145–152

Van den Eynde BJ and Boon T (1997) Tumor antigens recognized by T lymphocytes. *Int J Clin Lab Res* **27**: 81–86

Van den Eynde BJ and Van der Bruggen P (1997) T cell defined tumor antigens. *Curr Opin Immunol* **9**: 684–693

Van der Bruggen P, Traversari C, Chomez P, Lurquin C, de Plaen E, Van den Eynde B, Knuth A and Boon T (1991) A gene encoding an antigen recognized by cytolytic T-lymphocytes on a human melanoma. *Science* **254**: 1643–1646

Weynants P, Lethé B, Brasseur F, Marchand M and Boon T (1994) Expression of MAGE genes by non-small-cell lung carcinomas. *Int J Cancer* **56**: 826–829

Zuber M, Spagnoli GC, Kocher T, Luscher U, Schaefer C, Noppen C, Gudat F, Harder F and Heberer M (1997) Heterogeneity of melanoma antigen-1 (MAGE-1) gene and protein expression in malignant melanoma. *Eur Surg Res* **29**: 403–410

© 2000 Cancer Research Campaign
